# Modulation of PC1/3 activity by a rare double-site homozygous mutation

**DOI:** 10.3389/fped.2022.1026707

**Published:** 2022-10-31

**Authors:** Yanyan Ni, Xiangxiang Chen, Yi Sun, Jiarong Pan, Chao Tang, Tianming Yuan

**Affiliations:** ^1^ Department of Neonatology, The Children's Hospital, Zhejiang University School of Medicine, National Clinical Research Center for Child Health, Hangzhou, China; ^2^ National Clinical Research Center for Child Health of the Children's Hospital, Zhejiang University School of Medicine, Hangzhou, China

**Keywords:** proprotein convertase 1/3 deficiency, *PCSK1*, endocrine system diseases, metabolic acidosis, diarrhea, hypothyroidism, polyuria

## Abstract

**Objectives:**

Preprotein convertase 1/3 deficiency is a rare autosomal recessive disorder in which patients present with malabsorptive diarrhea and a series of symptoms of endocrine disorders such as polydipsia, reactive hypoglycemia, growth hormone deficiency, hypothyroidism, adrenal insufficiency, and early onset obesity. In its essence, pituitary hormone deficiency is caused by insufficient cleavage of pituitary prohormones. Here, we describe a female child with a rare double-site homozygous mutation in *PCSK1* (Proprotein convertase subtilisin/kexin-type 1) gene, and thereby intend to investigate the relationship between these novel mutation sites and changes in protein synthesis and function.

**Methods:**

We tested this patient's blood and urine fecal indicators of infection, blood electrolytes, and relevant endocrine hormone levels in the laboratory. Next Generation Sequencing was applied to screen the patient's DNA. Western Blot was performed to evaluate the mutant protein's expression. The enzymatic activity was measured as the rate of cleavage of a synthetic fluorogenic substrate in a specific solution.

**Results:**

We found that this patient presented shortly after birth with uncorrectable diarrhea and symptoms of metabolic acidosis with hypothyroidism. Next Generation Sequencing revealed that a rare double-site homozygous missense mutation, c.763G > A (p.G255R) and c.758C > T (p.S253L), were detected in exon 7 of *PCSK1* (Proprotein convertase subtilisin/kexin-type 1) gene on chromosome 5 of the patient. Western blotting revealed that there was no significant decrease in protein synthesis levels in the mutant phenotype compared to the wild type. Compared with WT type, the proteins expressed by the mutations showed a significant decrease in the enzyme activity towards the fluorescent substrates. However, neither the single site mutation p.S253L or p.G255R, nor the double-site mutation of both, all showed no significant differences from each other.

**Conclusions:**

These two missense mutations have not been reported before, and it is even rarer to find homozygous variation of two sites in one patient. This study identifies two novel mutations for the first time and further investigates the changes in protein synthesis and enzyme activity, providing a new pathway to continue to explore the pathogenesis of diseases associated with the function of PC1/3.

## Introduction

Proprotein convertase subtilisin/kexin type 1 gene (*PCSK1*), which encodes Prohormone convertase 1/3 (PC1/3), comprises 14 exons and is located on chromosome 5q15–21 in humans (1, 2). PC1/3 deficiency, an autosomal-recessive disorder caused by rare mutations in the *PCSK1* gene, whose main clinical manifestation is characterized by malabsorptive diarrhea, polyphagia with obesity, and a series of endocrinopathies, including growth hormone deficiency, adrenal insufficiency, central diabetes insipidus, central hypothyroidism, and hypogonadism (3). In the early stages of the condition, almost all patients require parenteral nutritional support, and diarrhea improves with age (3–5). The remaining endocrine function disorders usually necessitate symptomatic supplementation, such as thyroxine supplementation for hypothyroidism and sex hormone supplementation for hypogonadism (4). To our knowledge, 32 PC1/3 deficient patients have been reported in the literature. Among them, 28 (87.5%) patients had homozygous mutations and only 4 (12.5%) were compound heterozygotes (6–10). Depending on the location of the mutation site and the type of mutation, the impact on the final result of protein synthesis and enzyme activity frequently differs. In the homozygous missense mutation c.1777G > A (p. Gly593Arg), for instance, lack of conserved residues leads to synthesized products with low measured secretory activity, however PC1/3 can still be produced and identified (4); And the patient has the symptoms of malabsorptive diarrhea, hypoglycemia, central hypogonadism, obesity and central diabetes insipidus. The heterozygous missense/splice site mutation c.1777G > A (p. Gly593Arg)/c.620 + 4A > C resulted in the loss of conserved residue and intron donor sites, leading only pro-PC1/3 to be detected and PC1/3 production to be absent (11, 12), whilst the patient has the symptoms of malabsorptive diarrhea, hypoglycemia, central hypogonadism, obesity and growth hormone deficiency. In this study, we describe two novel missense mutant sites that haven't been reported before. Different from the cases reported previously, the case presented here is even more unusual as the infant has two homozygous mutation sites at the same time. We intend to look into the link between mutation sites and changes in protein synthesis and function in order to better understand the effects of the mutation. And we believe that it will give a new theoretical foundation and reference for the prevention and treatment of the *PCSK1* gene associated with PC1/3 deficiency.

## Materials and methods

### Subjects

This study was approved by the ethical Institutional Review Board (IRB) of the Children's Hospital, Zhejiang University School of Medicine (IRB number:2021-IRB-251).

### Targeted next generation sequencing(NGS) and data analysis

The patient's and her parents' genomic DNA were extracted from 2-ml peripheral blood samples using the QIAamp DNA Blood Mini Kit (Qiagen GmbH, Hilden, Germany). The DNA concentration and purity were measured using an Invitrogen Qubit dsDNA detection kit and a Qubit4 fluorometer (Carlsbad, CA, USA). The Agilent Sure Select Target Enrichment System (Agilent Technologies Inc., Santa Clara, CA, US) was used to produce an adapter-ligated library according to the manufacturer's instructions. An XT Inherited Disease Panel (cat No. 5190–7519, Agilent Technologies Inc.) containing 2,742 genes was used to create the capture library. The clusters were then produced using an Illumina cBot Station and sequenced on an Illumina HiSeq 2,500 System (Illumina Inc., San Diego, CA, US). Using human genome hg19 as the reference, alignment of sequence and repeated labeling were performed using BWM version 0.7.17 (http://bio-bwa.sourceforge.net/) and Picard bioinformatics software version 2.5.0 (https://broadinstitute.github.io/picard/) for biological analysis and interpretation. GATK 4.0.0.0 (https://gatk.broadinstitute.org/hc/en-us/sections/360007407851-4-0-0-0) and Samtools 1.8 (https://sourceforge.net/projects/samtools/files/samtools/1.8/) were used to identify mutation sites.

### Cell line and cell culture

Human embryonic kidney 293 cells (HEK293) were obtained from ATCC (Manassas, VA) and were cultured in high glucose DMEM (Hyclone, USA) supplemented with 10% (v/v) fetal bovine serum (FBS, Life Technologies, Inc., Grand Island, NY) as described previously (4, 13). Cells were maintained at 37°C with 5% CO2.

### Transient transfection of expression vectors & enzyme assay

A Flag-tag was inserted into the N-terminal of a wild-type human PC1/3 (NM 000439.5)-encoding plasmid, which was mutated at Ser 253 to encode Leu and Gly 255 to encode Arg by site-directed mutagenesis using the KOD-Plus Mutagenesis Kit (TOYOBO, Japan) and then sequenced in their entirety. There were no other mutations detected. The wild-type and mutant plasmids were transiently transfected into HEK293 cells using Lipofectamine 2,000 reagent (Invitrogen) as per the manufacturer's instructions. Two days later, the overnight conditioned Optimem (containing 100 mg/ml aprotinin) was assessed for protein expression by western blotting and enzymatic activity of secreted recombinant PC1/3 proteins present in conditioned medium was tested using a fluorogenic substrate, pERTKR-AMC (pyroGlutamate-Arginine-Threonine-Lysine-Arginine-aminomethylcoumarin, R&D Systems) as previously described (4, 13, 14).

### Western blot

Western blot was performed as described previously (15). Briefly, total protein extracts were prepared using lysis buffer (2% SDS, 10% glycerol, 50 mM Tris-HCl pH 6.8), and protein concentrations were determined by using a standard Bradford assay (Beyotime, Shanghai, China). 50 g of total protein was then subjected to SDS-PAGE followed by a transfer onto PVDF membranes (Millipore, Bedford, MA). Membranes were incubated with primary antibodies against Flag (MBL, Beijing, China) or GAPDH (Bioss, Beijing, China) at 4°C overnight followed by incubation in secondary antibodies (Beyotime, Shanghai, China) at room temperature for 2 h. Immunosignals were subsequently developed by using the Enhanced Chemiluminescence System. National Institutes of Health Image software (ImageJ, http://rsb.info.nih.gov/ij/) was used to quantify the immunoreactive bands, and the normalized antigen signals were calculated from Flag-derived and GAPDH-derived signals.

## Results

### Clinical phenotype description

The patient, a female infant born after 41 weeks of gestation, weighed 3150 g (−0.18 SD) and height 50 cm (0.83 SD). The patient admitted to the local hospital for management of severe diarrhea and metabolic acidosis within the third week of life, with polyuria and no abnormalities in urine and fecal routine and pathogen testing. Serum potassium was 2.2 mmol/L (normal 3.5–5.5 mmol/L), serum sodium was 152 mmol/L (normal 135–145 mmol/L), serum chloride was 145 mmol/L (normal 98–107 mmol/L), and the potential of hydrogen was 7.11 (normal 7.35–7.45). After stabilization in the local hospital, the infant was referred to our hospital on postnatal day 33 because of persistent diarrhea and acidosis. Blood inflammatory parameters were not significantly elevated, thyrotropin (TSH) 12.8 mIU/L (normal 0.35–4.94 mIU/L), free thyroxine (FT4) 9.81 pmol/L (normal 9.01–19.05 pmol/L) (31 days old). The patient was considered to have congenital hypothyroidism and was therefore given oral treatment with levo-thyroxine. Blood tests suggested carnitine deficiency, and levocarnitine was administered orally. The patient's karyotype is 46, XX.

When first admitted to our hospital, the infant weighed 3.32 kg (−1.94 SD) and height 52 cm (−0.79 SD). Except for dehydration, the physical examination revealed no visible abnormalities. Her blood tests revealed metabolic acidosis, hypernatremia, and hyperchloremia at the time of hospitalization. She was given 1 ml/kg/d of 10% potassium citrate orally for 5 days, as well as acid correction, rehydration, and other symptomatic care. Blood glucose levels were kept under control, and genetic metabolism tests in the blood and urine were normal. Levocarnitine was discontinued. TSH levels were within normal limits, but FT4 levels were constantly below 10 pmol/L. The ACTH level was slightly elevated (80 pg/ml), while the cortisol level was low (4.6 g/dl). While in the hospital, the baby developed polyuria, with a high volume of 8.2 ml/kg/h and a urine specific gravity of 1.008. After 23 days in the hospital, the infant was discharged with potassium citrate and weighed 3.75 kg (−2.25 SD) with a height of 52 cm (−1.52 SD). The patient had a cranial MRI at 2 months of age and no significant abnormalities were seen.

Follow-up: At 6 months of age, the infant was readmitted to our respiratory medicine department with “severe pneumonia.” She was 6 kg (−1.84 SD), 61 cm (−2.44 SD), and had been given additional food. She drank 120 ml of milk every two hours. She was still suffering from diarrhea and was taking potassium citrate orally at a rate of 4 ml/kg/d qid, as well as drinking roughly 750 ml of oral rehydration salts III daily. Blood gas monitoring revealed metabolic acidosis, and the FT4 level remained low (see brief summary of the patient's blood hormone levels in Table 1).

**Table 1:** Brief summary of the patient's hormonal evaluations and drug application.

Characteristic	Age						
	1 m	2 m	3 m	4 m	6 m	6.5 m	7 m
TSH (Ref 0.35–4.94 mIU/L^a^)	**8**.**553**	2.587	1.054	1.339	1.099	1.582	0.431
Free thyroxine (9.01–19.05 pmol/L)	**8**.**62**	10.73	11	**8**.**99**	9.35	**8**.**74**	**8**.**85**
Thyroxine (62.68–150.8 nmol/L)	**57**.**25**	80.09	76.31	**47**.**96**	65.22	65.62	**60**.**91**
levo-thyroxine (Dose per day/weight)	30 µg/ 3.32 kg	As before	As before	37.5 µg/ 4.6 kg	As before	40 µg/ 6 kg	50 µg/ 6 kg
ACTH (0–46 pg/ml)	**80**	-	50.7	-	43.3	17.9^b^	-
Cortisol (5–25 µg/dl)	**4**.**6**	-	7.64	-	10.7	7.76^b^	-
Aldosterone (50–900 ng/L)	492.39	-	-	-	-	-	-
Serum insulin (17.8–173 pmol/L)	-	78.5	-	-	-	-	128.5
Serum glucose (3.6–6.1 mmol/L)	-	4.9	-	-	-	-	4.7
IGF-1 (16) (34–344 ng/ml)	-	-	-	-	-	**30**.**6**	-
IGFBP-3 (16) (0.7–3.5 µg/ml)	-	-	-	-	-	1.26	-
GH (5–27 ng/ml)	-	-	5.02	-	-	-	-

Abnormal values are in bold.

-:Untested;

^a^ Reference value results from our laboratory;

^b^ Test at 8 am.

Ref, reference; m, months-of-age; TSH, thyroid stimulating hormone; ACTH, adrenocorticotropic hormone; IGF-1, insulin-like growth factor-1; IGFBP-3, insulin-like growth factor binding protein-3; GH, growth hormone.

Family Background: The patient's parents are middle-sized adults in good health, no history of chronic diarrhea as children, and no abnormalities of the thyroid or blood glucose levels during annual physical exams. The parents and family had no significant appearance deformities. Her older sister was in good condition. Other family members were not available for further genetic and phenotypic work-up.

### Identification of homozygous mutation in *PCSK1* by tNGS

**Figure 1:**
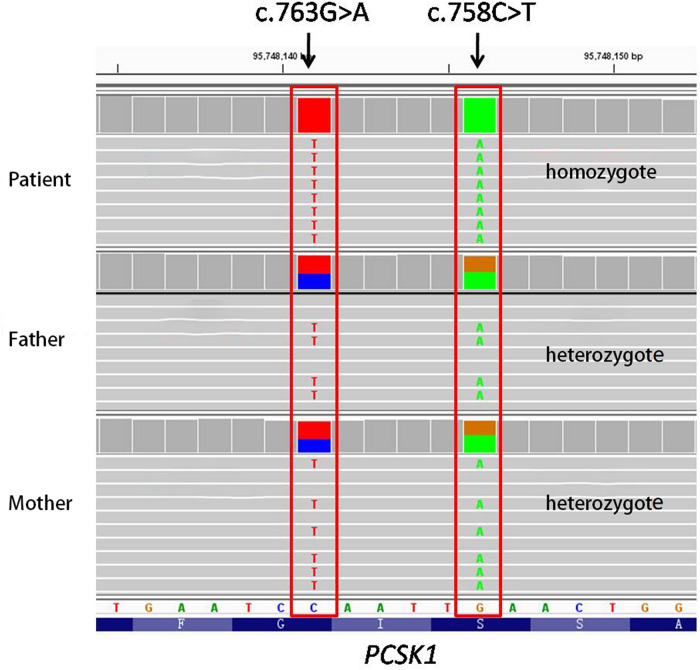
Next generation sequencing of this patient and her parents (The gene is reverse coded). NGS detected two homozygous missense mutations, c.758C > T (p.S253L) and c.763G > A (p.G255R), in exon 7 of the *PCSK1* gene of this patient. Both parents are simple heterozygotes since both mutations are located on the same strand (allele).

We used the trio model (i.e., both the patient and her parents were sequenced by second generation sequencing). The reported high-quality variants were not validated by Sanger sequencing. Next Generation Sequencing of this patient and her parents revealed a rare double-site homozygous missense mutation, c.763G > A (p.G255R) and c.758C > T (p.S253L) (NM_000439.5), were detected in exon 7 of *PCSK1* gene. Since the two mutations are located on the same strand (allele) as illustrated in Figure 1, both parents have a wild-type allele and a mutant allele (with 2 variants). Therefore, due to the recessive inheritance of the disease, the parents are simple heterozygotes with no clinical manifestations (https://doi.org/10.6084/m9.figshare.20764030.v2).

**Figure 2:**
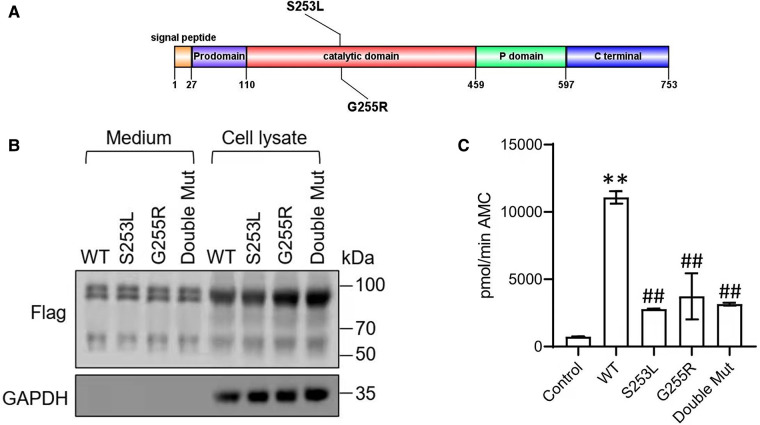
(**A**) Domain structure showing the novel S253L and G255L mutations within PC1/3. The PC1/3 exhibit an N-terminal signal peptide, followed by a prodomain, a catalytic-domain, a P-domain, and an enzyme-specific C-terminal segment. (**B**) Western blot of PC1/3 wild-type and mutant protein expression. HEK293 cells were transfected with Flag-tagged wildtype PC1/3 (WT) or PC1/3s containing novel mutations. Medium and cell lysate were subjected to western blot using an anti-Flag antibody. GAPHD was used as a loading control. (**C**) Enzymatic activity of wild-type PC1/3 (WT) or PC1/3s containing novel mutations. The enzymatic activities of secreted PC1/3 proteins in conditioned medium were assayed using a fluorogenic assay. **p < 0.01 vs. Control, ##*p* < 0.01 vs. WT; n = 6; error bar, SD.

The two variants c.763G > A (p.G255R) and c.758C > T (p.S253L) found in the patient were both missense mutations (Figure 2A). Searches of the ExAC, GnomAD and 1,000 Genomes databases did not show any relevant records and did not support the polymorphic sites. Protein function prediction by SIFT, Polyphen and Mutation Taster suggested that both two variants were “disease inducing”. No records were found in ClinVar, LOVD and HGMD databases. According to the ACMG guidelines, the two variants were rated as “Uncertain Significance”.

### Functional analysis and *in vitro* assessment

**Figure 3:**
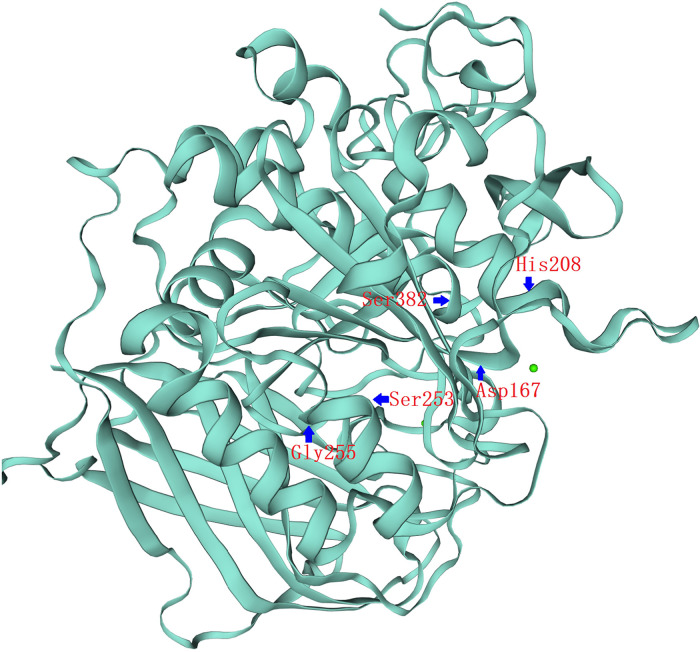
*PCSK1* catalytic domain 3D structure. 3D representation of the full protein and catalytic domain of PC1/3, based on the crystal structure of *PCSK1* using the Swiss model (swissmodel.expasy.org). Both variation sites of two residues, S253L&G255R, which closely nearby, are located in the exterior β-turn of the catalytic domain. The catalytic triad of Asp167, His208 and Ser382 residues have been labeled.

To confirm the functional significance of these two variants, we examined the activity of the wild type and mutant PC1/3 proteins in HEK293 cells. Western blotting revealed that both the single-mutant and double-mutant proteins had normal prodomain removal and were secreted efficiently from HEK293 cells (Figure 2B). All *PCSK1* mutations studied in this study destroyed the enzymatic activity of PC1/3 during examination in an HEK cell expression system. The missense mutants p.S253L and p.G255R could traverse the secretory pathway efficiently. The secreted protein exhibited variable enzyme activity against the fluorogenic substrate (Figure 2C). But compared with WT type, the proteins expressed by the mutations showed a significant decrease in the enzyme activity towards the fluorescent substrates. Neither the single site mutation p.S253L or p.G255R, nor the double-site mutation of both, all showed no significant differences from each other. In the PC1/3 3D model (Figure 3), both variation sites of two residues, which closely nearby, are located in the exterior β-turn of the catalytic domain (17), positions that may have a similar effect on protein folding.

## Discussion

Proprotein convertases (PCs) are a family of secreted mammalian serine endoproteases that convert inactive propeptides into physiologically active peptides because of their subtilisin-like catalytic domain (6, 18). There are seven closely related enzymes (furin, PC1/3, PC2, PC4, PACE4, PC5/6, and PC7), as well as two less related enzymes (PCSK8 and PCSK9) in the PC family (4, 13). PC1/3 is found in a variety of tissues, including gut endocrine cells, hypothalamic arcuate and paraventricular nuclei, monocytes, and pancreatic beta cells. Studies on PC1/3 knockout mice have clearly demonstrated that the production of specific peptides in such animals is decreased or abolished due to lack of processing *via* PC1/3 (19, 20). And it has been found that PC1/3 gene disruption results in severe developmental abnormalities (14). Growth retardation is observed in PC1/3-null mice, and adult mutant mice are approximately 60% the size of normal mice. Furthermore, analysis of many protein precursors known to be processed by PC1/3 revealed that these mice exhibit multiple abnormalities in a variety of hormone precursor processing events, including pituitary proopiomelanocortin (POMC), hypothalamic growth hormone releasing hormone (GHRH), proinsulin, and intestinal proglucagon. Therefore, patients with PC1/3 deficiency have been observed to suffer malabsorptive diarrhea, polyuria, reactive hypoglycemia, growth hormone deficiency, hypothyroidism, adrenal insufficiency, and early-onset obesity in varying degrees (17).

All the patients had malabsorptive diarrhea. There was no alleviation after ruling out the effects of infection, ion channel transport abnormalities, and various nutrients in the diet. Except for a few patients who showed evidence of modest villous atrophy, intestinal tube biopsies were conducted on some children and no substantial abnormalities were discovered (4). Normal chromogranin A staining was found in the intestinal tissues, however, neither PC1/3 nor PC2 expression was found (4, 5, 21).

Most children's diarrhea symptoms improved with age, and they exhibited hyperappetence and rapid weight gain around the age of 12 to 18 months (4, 22). Stijnen et al. (1) discovered a link between the *PCSK1* SNPs rs6232 and rs6234-rs6235 and obesity in their study. Body mass index (BMI) in individuals with PC1/3 deficiency rises gradually after the age of two years, however in many patients, weight growth and short stature happen concurrently, implying that the rise in BMI is not simply due to weight gain (4, 5). Another mouse model of PC1/3 deficiency which has a missense mutation in the PC1/3 catalytic domain (N222D), leading to obesity with abnormal proinsulin processing and multiple endocrine deficiencies (23). One patient with PC1/3 deficiency was diagnosed with severe childhood obesity (12). PC1/3 is thought to play a crucial part in the production of numerous hormones, and so may contribute to obesity risk by involving in appetite regulation, such as feeding behavior and thermogenesis (1, 24). In a series of studies investigating the possible role of leptin in the regulation of neuropeptide processing, it was reported the regulatory role of leptin on PC1/3 in the paraventricular nucleus arcuate nucleus of the hypothalamus, and the nucleus of the solitary tract (25, 26).

A new case of PC1/3 deficiency with characteristic symptoms such as severe congenital diarrhea, polyuriaand central hypothyroidism is reported in this article. NGS detected two new missense mutations in *PCSK1*, c.763G > A (p. G255R) and c.758C > T (p. S253L). Patients with PC1/3 deficiency have been reported to exhibit multiple hormonal insufficiencies. The form and severity of hormonal insufficiency vary from patient to patient with PC1/3 deficiency. To date, there have been 32 cases of *PCSK1* deficiency recorded so far, with patients presenting a varied range of symptoms (6, 8, 9). The first patient with a *PCSK1* mutation was reported by O'Rahilly et al. in 1995 (11); of total 33 patients diagnosed (including the one we reported) (see Table 2), all presented with malabsorptive diarrhea before the age of 3 years, 60.6% (20/33) in males and 39.4% (13/33) in females. 87.9% (29/33) had onset in the neonatal period. Also, 72.7% (24/33) presented with polydipsia and polyuria or were diagnosed with central uremia, 69.7% (23/33) were diagnosed with early-onset obesity, 48.5% (16/33) had hypocorticism, 54.5% (18/33) had central hypothyroidism, 33.3% (11/33) had reactive hypoglycemia, and 33.3% (11/33) were diagnosed with growth hormone deficiency, 30.3% (10/33) were found to have hypogonadotrophic hypogonadism, and 4 died before 2 years of age (12.1%). There are significant similarities in the clinical symptoms of patients with the same site variant based on the variant sites and clinical manifestations of the documented instances thus far. For example, two patients with the c.1213C > T variation developed diarrhea, hypoglycemia, obesity, and polyuria or diabetic insipidus. Two patients with the c.1_2delATinsTA variant had diarrhea, obesity, hypocorticism and hypothyroidism; Two patients with the c.1312C > T variant had diarrhea, obesity and diabetic insipidus; Two patients with the c.500A > C (p. D167A) variant had diarrhea, obesity, polyuria or diabetic insipidus. Both of the two patients with the c.500A > C (p. D167A) variant experienced diarrhea, obesity, polyuria, hypocorticism, growth hormone insufficiency, and hypothyroidism, with minor variances attributable to age-related variations in gonadal and growth hormone action. However, except for diarrhea, the three patients who all had the c.927C > G variant and the two patients who all had the c.1350_1353del (p. D451fs) mutation had little in common. It's difficult to adequately assess the association between genotype and phenotype due to the small number of instances reported so far.

**Table 2:** Overview of PC1/3-deficient patients: genotype and clinical phenotypes.

ID	Refrence No.	Sex	Consan-guinity	Genotype (Mutation)	Age presented	Phenotype	present situation
1	(11, 12)	F	NA	c.1777G > A (p.G593R)/ c.620 + 4A > C, HeM	within 3y	①②③⑥⑦⑨	survived
2	(3)	F	No	c.748G > T (p.E250*)/c.638_640delCAG (p.A213del), HeM	3d	①②③⑥	Died at 18 m^a^
3	(22)	M	Yes	c.920C > T (p.S307L), HoM	8d	①③⑤	survived
4	(27)	M	NA	c.1024delT (p. W342GfsX92)/c.-775209_*59002del, HeM	3d	①②③④⑥⑧⑨	survived
5	(4)	M	Yes	c.1777G > A (p.G593R), HoM	3wk	①②③④⑥⑧⑨	survived
6	(4)	M	Yes	c.625G > A (p.G209R)/c.772C > A (p.P258T), HeM	7d	①②⑤	survived
7	(4, 28)	F	Yes	c.1095 + 1G > T (intron donor site loss), HoM	7d	①③④⑥⑦⑧⑨	survived
8	(4)	M	Yes	c.1009C > T (p.Q337*), HoM	7d	①	Died at 8 m^b^
9	(4, 29)	M	No	c.1213C > T (p.R405*), HoM	7d	①②③⑤⑥	survived
10	(4, 29)	M	No	c.1213C > T (p.R405*), HoM	7d	①②③④⑥⑨	survived
11	(4, 29)	M	Yes	c.1_2delATinsTA (p.M1*), HoM	2wk	①③④⑥⑦⑧	survived
12	(4, 29)	M	Yes	c.1_2delATinsTA (p.M1*), HoM	7d	①③⑥⑦⑧	survived
13	(4)	F	Yes	c.1095 + 1G > A, HoM	4wk	①②⑤⑧	Died at 15 m^b^
14	(4)	M	Yes	c.1349_1352del (p.V450Vfs*1), HoM	8wk	①⑤⑧	survived
15	(4)	M	Yes	c.1643T > C (p.F548S), HoM	3wk	①②③④⑥	survived
16	(4)	F	Yes	c.693C > G (p.Y231*), HoM	2wk	①②③④⑥⑧	survived
17	(4)	M	No	c.1269C > A (p.N423K), HoM	2wk	①③④⑦⑧⑨	survived
18	(5)	M	Yes	c.1029C > A (p.Y343*), HoM	<3wk	①④⑨	survived
19	(21)	F	Yes	c.927C > G (p.N309K), HoM	8d	①③④⑦⑧	survived
20	(21)	F	Yes	c.927C > G (p.N309K), HoM	within 1mo	①⑧	Died at 5 m^c^
21	(21)	M	Yes	c.927C > G (p.N309K), HoM	within 1mo	①	survived
22	(30)	F	Yes	c.544-2A > G, HoM	<2y	①③④	survived
23	(31)	M	Yes	c.1312C > T (p.R438*), HoM	1d	①③④⑦	survived
24	(31)	M	Yes	c.1312C > T (p.R438*), HoM	2wk	①③④	survived
25	(32)	M	Yes	c.679del (p.V227Lfs*12), HoM	7d	①②③⑧	survived
26	(6)	M	Yes	c.595C > T (p.R199*), HoM	3d	①③④⑥⑦⑧⑨	survived
27	(10)	F	No	c.685G > T (p.V229F), HoM	12d	①③⑩	survived
28	(8)	F	Yes	c.1350_1353del (p.D451fs), HoM	13d	①⑥⑦⑧	survived
29	(8)	F	Yes	c.1350_1353del (p.D451fs), HoM	6mo	①③⑤	survived
30	(9)	F	Yes	c.500A > C (p.D167A), HoM	1d	①③④⑥⑦⑧	survived
31	(9)	M	Yes	c.500A > C (p.D167A), HoM	3d	①③④⑥⑦⑧	survived
						⑨	
32	(7)	M	Yes	c.1034A > C (p.E345A), HoM	1wk	①④⑤⑥⑧⑨	survived
33		F	NA	[c.758C > T (p.S253L); c.763G > A (p.G255R)], HoM	17d	①⑤⑧	survived

①: Malabsorptive Diarrhea; ②: Postprandial Hypoglycemia; ③: Obesity; ④: Central Diabetes Insipidus; ⑤: Polydipsia and polyuria; ⑥: Central Hypercortisolism; ⑦: Growth hormone deficiency; ⑧: Central Hypothyroidism; ⑨: Hypogonadism; ⑩: Diabetes.

^a^ Died of cardiac and respiratory arrest (cause unknown).

^b^ Died of sepsis due to central venous infection.

^c^ Died of intractable epilepsy (another genetic disorder in the family).

F, female; M, male; NA, not assessed; HeM, heterozygous mutation; HoM, homozygous mutation; d (days-of-age); wk (weeks-of-age); m (months-of-age).

The patient we report had a height and weight within the normal range at birth. But by the time she was 6 months old, both measurements had fallen almost two standard deviations behind. Growth hormone level was at the lower limit of the reference range (5.02 ng/ml) at 2 months of age, and insulin-like growth factor-1 (IGF-1) level was slightly lower (30.6 ng/ml) at 6 months (Table 1). The patient must be constantly watched for growth and development going forward because of the increased risk of growth hormone insufficiency. It is important to keep a close watch on the growth hormone-insulin growth factor (GH-IGF) axis and to perform growth hormone stimulation tests at the proper time.

Shortly after birth, it was discovered that this patient had low free thyroxine (FT4) and high thyrotropin (TSH) levels. Typically, it is presumed that low FT4 secretion is what causes increased TSH reactivity, and hypothyroidism needs to be taken into consideration. After taking levothyroxine orally, the patient's total T4 and FT4 levels remained low, but TSH levels were abnormally normal, thyroid hormone-binding globulin was consistently within the normal range, and thyroid ultrasonography did not show any ectopic or size abnormalities. Less TRH is produced when the hypothalamus is injured, which decreases the release of TSH, Triiodothyronine (T3), and T4, resulting in central hypothyroidism. Because of this, we assumed that the patient had this problem. Although we gave the patient what we believe to be an appropriate dose of levothyroxine as a supplement, the patient's oral medication absorption was probably hampered by the long-term presence of chronic malabsorptive diarrhea, leading to consistently lower than anticipated free thyroxine levels in the blood. We'll follow up with dose modifications and keep a close eye on the patient's thyroid hormone levels.

The patient was admitted with a little increased adrenocorticotropic hormone (ACTH) level (80 pg/ml) and a slightly low cortisol level (4.6 g/dl), but her clinical symptoms—hypokalemia and hypernatremia—did not point to a mineralocorticoid insufficiency. Aldosterone level was also within the normal range. We adopted expectant therapy rather than beginning hydrocortisone medication because the patient's electrolyte imbalance was alleviated after rehydration therapy, blood glucose monitoring was always within normal ranges, and vital signs were stable. We randomly rechecked ACTH and cortisol levels at 3 months of age (when the patient was informed of the NGS test result) and 6 months of age, both of which were within the normal range, since infants under 3 months of age do not have a regular sleep pattern and do not have a circadian pattern of cortisol secretion. When the patient's cortisol was checked at 8:00 am at 6.5 months old, it was within the usual range, and the ACTH level was not abnormally elevated. During hospitalization and outpatient follow-up, blood glucose and serum insulin levels were within normal ranges.

Throughout her hospital stay, the patient's output was consistently higher than 70% of her intake.

Despite the low urine gravity of the patient (as low as 1.008), the presence of diabetes insipidus cannot yet be diagnosed. The patient's diarrhea and polyuria persisted during the outpatient follow-up. It is quite regrettable that for a variety of reasons, we were unable to finish the water-deprivation test and the ACTH stimulation test. Although we are unable to confirm central adrenal insufficiency and diabetes insipidus in our patient at this time to thoroughly evaluate pituitary function, we will complete these relevant endocrine work-ups in the future during long-term follow-up.

PC1/3 comprise four domain parts: a prodomain, a catalytic domain, a P-domain and a C-terminal domain (33–36) (Figure 2A). After initially synthesized as a zymogen, PC1/3 is cleaved autocatalytically in the endoplasmic reticulum rapidly (37). After being produced as a 97-kDa zymogen, PC1/3 is successively autocatalytically converted to 87-kDa (38) and to the smaller 74/66-kDa active form in the endoplasmic reticulum. The latter is less steady than the former but more active (37–39). *In vivo* studies have also revealed increased activity of the 66-kDa variant and only when the 66-kDa form further cleaves the C-terminal peptide, this convertase can be finally free to act in trans on the majority of its substrates (40). Mutations or deletions in convertase propeptides have long been known to block autocatalytic processing in the endoplasmic reticulum (33, 41). Previous research has also suggested that structural interactions between the P domain and the catalytic domain play a significant role in determining the pH optimum and specific activity (42).

The catalytic domain is a highly conserved domain in human pro-protein serine convertases, and it is part of the catalytic triad Ser/His/Asp catalytic, which is vital in catalyzing peptide bond hydrolysis (43). According to conformational research based on the crystal structure of *PCSK1*, the affected amino acids, Ser253 and Gly255, are located in the exterior β-turn of the catalytic domain (Figure 3). As a result, missense mutations on Ser253 or Gly255 may not affect enzymatic activity as strongly as previously described missense variants closer to the highly conserved catalytic triad Ser/His/Asp (4, 9). Also, the catalytic domains of the PC1/3 protein, which have a high degree of conservation, are located between sites 158 and 442 while the disulfide bond region is located between sites 225 and 374. It is very likely that a pure mutation of these two locations may alter the production of disulfide bonds, impairing PC1/3 function. Meanwhile, the degree of decrease in enzyme activity produced by either the single-site homozygous mutation or the double-site homozygous mutation in p. S253L or p. G255R was similar and did not differ significantly, according to the results of the enzyme activity experiment. Given the close proximity of S253 and G255, missense mutations at these two positions are very likely to have the same effect. This could explain why the enzyme activity of the patient did not differ significantly between the two loci variants present at the same time compared to the single locus variant.

## Conclusion

PC1/3 deficiency is a disease that affects the secretion of multiple hormones and currently has no effective cure. In clinical practice, patients with affected hormone secretion and persistent non-infectious, malabsorptive diarrhea should be alerted to the disease and genetic testing should be performed if necessary to facilitate the diagnosis. The subsequent diarrhea, hormonal changes and growth of the child in this case are yet to be followed up. This study identifies two novel mutations for the first time and further investigates the changes in protein synthesis and enzyme activity, providing a new pathway to continue to explore the pathogenesis of diseases associated with the function of PC1/3.

## Data Availability

The datasets presented in this study can be found in online repositories. The names of the repository/repositories and accession number(s) can be found in the article/Supplementary Material.

## References

[1] Stijnen P Ramos-Molina B O'Rahilly S Creemers JW. PCSK1 mutations and human endocrinopathies: from obesity to gastrointestinal disorders. Endocr Rev. (2016) 37(4):347–71. 10.1210/er.2015-1117 27187081

[2] Seidah NG Mattei MG Gaspar L Benjannet S Mbikay M Chrétien M. Chromosomal assignments of the genes for neuroendocrine convertase PC1 (NEC1) to human 5q15-21, neuroendocrine convertase PC2 (NEC2) to human 20p11.1-11.2, and furin (mouse 7[D1-E2] region). Genomics. (1991) 11(1):103–7. 10.1016/0888-7543(91)90106-o 1765368

[3] Jackson RS Creemers JW Farooqi IS Raffin-Sanson ML Varro A Dockray GJ Small-intestinal dysfunction accompanies the complex endocrinopathy of human proprotein convertase 1 deficiency. J Clin Invest. (2003) 112(10):1550–60. 10.1172/jci18784 14617756 PMC259128

[4] Martín MG Lindberg I Solorzano-Vargas RS Wang J Avitzur Y Bandsma R Congenital proprotein convertase 1/3 deficiency causes malabsorptive diarrhea and other endocrinopathies in a pediatric cohort. Gastroenterology. (2013) 145(1):138–48. 10.1053/j.gastro.2013.03.048 23562752 PMC3719133

[5] Yourshaw M Solorzano-Vargas RS Pickett LA Lindberg I Wang J Cortina G Exome sequencing finds a novel PCSK1 mutation in a child with generalized malabsorptive diarrhea and diabetes insipidus. J Pediatr Gastroenterol Nutr. (2013) 57(6):759–67. 10.1097/MPG.0b013e3182a8ae6c 24280991 PMC4170062

[6] Pépin L Colin E Tessarech M Rouleau S Bouhours-Nouet N Bonneau D A new case of PCSK1 pathogenic variant with congenital proprotein convertase 1/3 deficiency and literature review. J Clin Endocrinol Metab. (2019) 104(4):985–93. 10.1210/jc.2018-01854 30383237

[7] Aerts L Terry NA Sainath NN Torres C Martin MG Ramos-Molina B Novel homozygous inactivating mutation in the PCSK1 gene in an infant with congenital malabsorptive diarrhea. Genes (Basel). (2021) 12(5). 10.3390/genes12050710 34068683 PMC8151971

[8] Ahmed ABM Alsaleem BMR. Enteroendocrine dysfunction in two Saudi sisters. Case Rep Gastroenterol. (2021) 15(1):290–95. 10.1159/000511761 33790717 PMC7989775

[9] Duclaux-Loras R Bourgeois P Lavrut PM Charbit-Henrion F Bonniaud-Blot P Maudinas R A novel mutation of PCSK1 responsible for PC1/3 deficiency in two siblings. Clin Res Hepatol Gastroenterol. (2021) 45(6):101640. 10.1016/j.clinre.2021.101640 33662777

[10] Karacan Kucukali G Savas Erdeve S Cetinkaya S Keskin M Bulus AD Aycan Z. A case of prohormone convertase deficiency diagnosed with type 2 diabetes. Turk Arch Pediatr. (2021) 56(1):81–4. 10.14744/TurkPediatriArs.2020.36459 34013237 PMC8114606

[11] O'Rahilly S Gray H Humphreys PJ Krook A Polonsky KS White A Brief report: impaired processing of prohormones associated with abnormalities of glucose homeostasis and adrenal function. N Engl J Med. (1995) 333(21):1386–90. 10.1056/nejm199511233332104 7477119

[12] Jackson RS Creemers JW Ohagi S Raffin-Sanson ML Sanders L Montague CT Obesity and impaired prohormone processing associated with mutations in the human prohormone convertase 1 gene. Nat Genet. (1997) 16(3):303–6. 10.1038/ng0797-303 9207799

[13] Pickett LA Yourshaw M Albornoz V Chen Z Solorzano-Vargas RS Nelson SF Functional consequences of a novel variant of *PCSK1*. PLoS One. (2013) 8(1):e55065. 10.1371/journal.pone.0055065 23383060 PMC3557230

[14] Hoshino A Kowalska D Jean F Lazure C Lindberg I. Modulation of PC1/3 activity by self-interaction and substrate binding. Endocrinology. (2011) 152(4):1402–11. 10.1210/en.2010-1170 21303942 PMC3060626

[15] Jin M Xu S Li J Li L Tang C. Role of ARID1A in the regulation of human trophoblast migration and invasion. Reprod Sci. 29(8):2363–73. 10.1007/s43032-021-00686-0 34255312

[16] Elmlinger MW Kühnel W Weber MM Ranke MB. Reference ranges for two automated chemiluminescent assays for serum insulin-like growth factor I (IGF-I) and IGF-binding protein 3 (IGFBP-3). Clin Chem Lab Med. (2004) 42(6):654–64. 10.1515/cclm.2004.112 15259383

[17] Henrich S Lindberg I Bode W Than ME. Proprotein convertase models based on the crystal structures of furin and kexin: explanation of their specificity. J Mol Biol. (2005) 345(2):211–27. 10.1016/j.jmb.2004.10.050 15571716

[18] Siezen RJ Leunissen JA. Subtilases: the superfamily of subtilisin-like serine proteases. Protein Sci. (1997) 6(3):501–23. 10.1002/pro.5560060301 9070434 PMC2143677

[19] Berman Y Mzhavia N Polonskaia A Devi LA. Impaired prohormone convertases in Cpe(fat)/Cpe(fat) mice. J Biol Chem. (2001) 276(2):1466–73. 10.1074/jbc.M008499200 11038363

[20] Zhu X Zhou A Dey A Norrbom C Carroll R Zhang C Disruption of PC1/3 expression in mice causes dwarfism and multiple neuroendocrine peptide processing defects. Proc Natl Acad Sci U S A. (2002) 99(16):10293–8. 10.1073/pnas.162352599 12145326 PMC124907

[21] Wilschanski M Abbasi M Blanco E Lindberg I Yourshaw M Zangen D A novel familial mutation in the PCSK1 gene that alters the oxyanion hole residue of proprotein convertase 1/3 and impairs its enzymatic activity. PLoS One. (2014) 9(10):e108878. 10.1371/journal.pone.0108878 25272002 PMC4182778

[22] Farooqi IS Volders K Stanhope R Heuschkel R White A Lank E Hyperphagia and early-onset obesity due to a novel homozygous missense mutation in prohormone convertase 1/3. J Clin Endocrinol Metab. (2007) 92(9):3369–73. 10.1210/jc.2007-0687 17595246

[23] Lloyd DJ Bohan S Gekakis N. Obesity, hyperphagia and increased metabolic efficiency in Pc1 mutant mice. Hum Mol Genet. (2006) 15(11):1884–93. 10.1093/hmg/ddl111 16644867

[24] Creemers JW Choquet H Stijnen P Vatin V Pigeyre M Beckers S Heterozygous mutations causing partial prohormone convertase 1 deficiency contribute to human obesity. Diabetes. (2012) 61(2):383–90. 10.2337/db11-0305 22210313 PMC3266396

[25] Sanchez VC Goldstein J Stuart RC Hovanesian V Huo L Munzberg H Regulation of hypothalamic prohormone convertases 1 and 2 and effects on processing of prothyrotropin-releasing hormone. J Clin Invest. (2004) 114(3):357–69. 10.1172/jci21620 15286802 PMC484982

[26] Perello M Stuart RC Nillni EA. Differential effects of fasting and leptin on proopiomelanocortin peptides in the arcuate nucleus and in the nucleus of the solitary tract. Am J Physiol Endocrinol Metab. (2007) 292(5):E1348–57. 10.1152/ajpendo.00466.2006 17227963

[27] Frank GR Fox J Candela N Jovanovic Z Bochukova E Levine J Severe obesity and diabetes insipidus in a patient with PCSK1 deficiency. Mol Genet Metab. (2013) 110(1-2):191–4. 10.1016/j.ymgme.2013.04.005 23800642 PMC3759845

[28] Gönç EN Özön A Alikaşifoğlu A Kandemir N. Long-term follow-up of a case with proprotein convertase 1/3 deficiency: transient diabetes mellitus with intervening diabetic ketoacidosis during growth hormone therapy. J Clin Res Pediatr Endocrinol. (2017) 9(3):283–87. 10.4274/jcrpe.3986 28588004 PMC5596812

[29] Bandsma RH Sokollik C Chami R Cutz E Brubaker PL Hamilton JK From diarrhea to obesity in prohormone convertase 1/3 deficiency: age-dependent clinical, pathologic, and enteroendocrine characteristics. J Clin Gastroenterol. (2013) 47(10):834–43. 10.1097/MCG.0b013e3182a89fc8 24135795 PMC3842618

[30] Härter B Fuchs I Müller T Akbulut UE Cakir M Janecke AR. Early clinical diagnosis of PC1/3 deficiency in a patient with a novel homozygous PCSK1 splice-site mutation. J Pediatr Gastroenterol Nutr. (2016) 62(4):577–80. 10.1097/mpg.0000000000001018 26488123

[31] Kumar G Nair R Sohal APS. Proprotein convertase 1/3 deficiency. Indian J Pediatr. (2018) 85(4):320–21. 10.1007/s12098-017-2479-x 28965329

[32] Distelmaier F Herebian D Atasever C Beck-Woedl S Mayatepek E Strom TM Blue diaper syndrome and PCSK1 mutations. Pediatrics. (2018) 141(Suppl 5):S501–s05. 10.1542/peds.2017-0548 29610180

[33] Creemers JW Vey M Schäfer W Ayoubi TA Roebroek AJ Klenk HD Endoproteolytic cleavage of its propeptide is a prerequisite for efficient transport of furin out of the endoplasmic reticulum. J Biol Chem. (1995) 270(6):2695–702. 10.1074/jbc.270.6.2695 7852339

[34] Zhou A Martin S Lipkind G LaMendola J Steiner DF. Regulatory roles of the P domain of the subtilisin-like prohormone convertases. J Biol Chem. (1998) 273(18):11107–14. 10.1074/jbc.273.18.11107 9556596

[35] Van de Ven WJ Roebroek AJ Van Duijnhoven HL. Structure and function of eukaryotic proprotein processing enzymes of the subtilisin family of serine proteases. Crit Rev Oncog. (1993) 4(2):115–36. 8420571

[36] Seidah NG Day R Marcinkiewicz M Chrétien M. Precursor convertases: an evolutionary ancient, cell-specific, combinatorial mechanism yielding diverse bioactive peptides and proteins. Ann N Y Acad Sci. (1998) 839:9–24. 10.1111/j.1749-6632.1998.tb10727.x 9629127

[37] Lindberg I. Evidence for cleavage of the PC1/PC3 pro-segment in the endoplasmic reticulum. Mol Cell Neurosci. (1994) 5(3):263–8. 10.1006/mcne.1994.1030 8087424

[38] Zhou Y Lindberg I. Enzymatic properties of carboxyl-terminally truncated prohormone convertase 1 (PC1/SPC3) and evidence for autocatalytic conversion. J Biol Chem. (1994) 269(28):18408–13. 10.1016/S0021-9258(17)32323-2 8034588

[39] Rufaut NW Brennan SO Hakes DJ Dixon JE Birch NP. Purification and characterization of the candidate prohormone-processing enzyme SPC3 produced in a mouse L cell line. J Biol Chem. (1993) 268(27):20291–8. 10.1016/S0021-9258(20)80727-3 8376387

[40] Ozawa A Peinado JR Lindberg I. Modulation of prohormone convertase 1/3 properties using site-directed mutagenesis. Endocrinology. (2010) 151(9):4437–45. 10.1210/en.2010-0296 20610561 PMC2940488

[41] Muller L Cameron A Fortenberry Y Apletalina EV Lindberg I. Processing and sorting of the prohormone convertase 2 propeptide. J Biol Chem. (2000) 275(50):39213–22. 10.1074/jbc.M003547200 10995742

[42] Henrich S Cameron A Bourenkov GP Kiefersauer R Huber R Lindberg I The crystal structure of the proprotein processing proteinase furin explains its stringent specificity. Nat Struct Biol. (2003) 10(7):520–6. 10.1038/nsb941 12794637

[43] Page MJ Di Cera E. Serine peptidases: classification, structure and function. Cell Mol Life Sci. (2008) 65(7-8):1220–36. 10.1007/s00018-008-7565-9 18259688 PMC11131664

